# Fecal Microbial Transplantation and Its Potential Application in Cardiometabolic Syndrome

**DOI:** 10.3389/fimmu.2019.01341

**Published:** 2019-06-14

**Authors:** Avner Leshem, Nir Horesh, Eran Elinav

**Affiliations:** ^1^Immunology Department, Weizmann Institute of Science, Rehovot, Israel; ^2^Department of Surgery, Tel Aviv Sourasky Medical Center, Tel Aviv, Israel; ^3^Department of General Surgery B and Organ Transplantation, Sheba Medical Center, Ramat Gan, Israel; ^4^Cancer-Microbiome Division, Deutsches Krebsforschungszentrum (DKFZ), Heidelberg, Germany

**Keywords:** FMT, fecal microbiome transplantation, cardiometabolic disease, microbiome, microbiota

## Abstract

Newly revealed links between inflammation, obesity, and cardiometabolic syndrome have created opportunities to try previously unexplored therapeutic modalities in these common and life-risking disorders. One potential modulator of these complex disorders is the gut microbiome, which was described in recent years to be altered in patients suffering from features of cardiometabolic syndrome and to transmit cardiometabolic phenotypes upon transfer into germ-free mice. As a result, there is great interest in developing new modalities targeting the altered commensal bacteria as a means of treatment for cardiometabolic syndrome. Fecal microbiota transplantation (FMT) is one such modality in which a disease-associated microbiome is replaced by a healthy microbiome configuration. So far clinical use of FMT has been overwhelmingly successful in recurrent Clostridium difficile infection and is being extensively studied in other microbiome-associated pathologies such as cardiometabolic syndrome. This review will focus on the rationale, promises and challenges in FMT utilization in human disease. In particular, it will overview the role of the gut microbiota in cardiometabolic syndrome and the rationale, experience, and prospects of utilizing FMT treatment as a potential preventive and curative treatment of metabolic human disease.

## Cardiometabolic Syndrome

Cardiometabolic syndrome (also termed “Metabolic syndrome”) consists of the co-occurrence of a cluster of pathogenically-associated metabolic disorders including obesity, insulin resistance, non-alcoholic fatty liver disease, hypertension, and hypercholesterolemia. When present, the combined effect of these disorders significantly increases the risk of developing cardiovascular disease and type 2 Diabetes Mellitus (TIIDM) (1). It is estimated that 22% of the adult population in the US suffers from cardiometabolic syndrome and prevalence is on the rise, especially in patients older than 60 years old where prevalence is 43.5% (2–4). The clinical implications of cardiometabolic syndrome are predominantly related to the increased risk of developing cardiovascular complications of atherosclerosis and micro- or macrovascular complications of TIIDM. Several studies estimated that the relative risk (RR) for developing cardiovascular disease is double than the general population (RR = 1.53–2.18) in patients suffering from cardiometabolic syndrome (5–7), coupled with an increase in all-cause mortality (RR = 1.27–1.6). In addition, the relative risk of developing TIIDM was significantly higher in patients suffering from other features of cardiometabolic disease (RR = 3.53–5.17) as compared to the general population (8).

In addition to the above metabolic and cardiovascular complications, cardiometabolic syndrome is associated with an aberrant inflammatory and coagulative response, including increased levels of proinflammatory markers including C-Reactive Protein, interleukin (IL)-6, and plasminogen activator inhibitor (PAI)-1 (9–13). Although these inflammatory and prothrombotic markers were shown to be associated with an increased risk of cardiovascular disease and TIIDM, the exact mechanisms by which they act to increase this risk remain unclear. In recent years there is increasing evidence that these inflammatory processes might be related to an imbalance in the immunologic response of the host in relation to host microbiota (14–16).

## The Gut Microbiota

The commensal gut microbiota is a “signaling hub” in many physiological functions of the mammalian host and especially in host's metabolism (17). A staggering amount of scientific evidence was gathered on the potential role of the commensal microbiota in influencing human health (18, 19) and a variety of multi-factorial diseases like Inflammatory bowel disease (IBD) (20, 21), Irritable bowel syndrome (IBS) (22, 23), and gastrointestinal cancer (24–26). The gut microbiota was also shown to have significance in non-gastrointestinal conditions such as cardiometabolic (27, 28), neurologic (29), and even psychiatric disorders (30–32).

There is great need to deepen the mechanistic understanding of commensal microbiota along with their function, secreted molecules repertoire, and their precise impacts on the host. An approach which favors mechanisms over correlations is much more likely to illuminate therapeutic targets for preventing or treating microbiota-associated diseases by means of antibiotics, prebiotics, probiotics, and fecal microbial transplants. While antibacterial treatment (such as antibiotics) has profound effects on the gut microbiota (33), it is non-specific and associated with the emergence of resistant strains, which precludes it from being a safe long-term microbiome intervention in chronic disease. Some nutritional interventions are known to affect cardiometabolic diseases including TIIDM and obesity by targeting the gut microbiota (34–36). Examples of dietary interventions include prebiotics, substances that include dietary fibers and oligosaccharides, which were suggested to have a potential beneficial effect on human health that is also correlated with alternations in gut microbiota (37). Probiotics, available in multiple food formulations, are aimed at modulating the host and its microbiome. However, there is contradicting evidence as to the beneficial effect that probiotics have on human health, with large-scale, non-industry sponsored high-quality clinical trials still missing for the majority of claimed indications (38, 39). Other experimental microbiome interventions include “postbiotic” treatment, utilizing microbiome-modulated metabolites as means of treatment (40, 41). Phage therapy is emerging as a promising pathobiont-eradicating therapeutic modality (42–44). Although bacteriophages, i.e., viruses that exclusively infect specific bacteria, are mostly studied in the context of treating antibiotic-resistant infections (45–47), their potential to specifically target bacterial strains may be harnessed to manipulate the gut microbiota to a more metabolically healthy composition (48, 49). Additionally, an intervention targeting the host interface of the host-microbiome gut niche may diminish some deleterious inflammatory consequences of obesity and diabetes (50).

## Fecal Microbiota Transplantation

Replacement of the indigenous microbiome of patients afflicted with microbiota-associated diseases with a “healthy” microbial configuration was termed “Fecal Microbiota Transplantation” (FMT) and is emerging as a new therapeutic method for various microbiota-associated pathological conditions. The process involves the collection of filtered stools collected from either a healthy donor or from the recipient himself (autologous FMT) at a time point prior to initiation of disease and associated dysbiosis and its instillation into the intestinal tract of a patient suffering from a certain medical condition. In most of the below review, we will refer to FMT performed by microbiome transfer across different individuals. So far, the therapeutic efficacy of FMT has been overwhelmingly significant in *Clostridium difficile* infection and recently in some studies in Ulcerative Colitis, but is proving to be much more challenging in other complex human conditions.

The use of feces transferred from healthy donors in treating patients suffering from diarrhea dates back to ancient Chinese medicine, nearly 1700 years ago (51). Modern era use of FMT was first described by Eiseman et al. (52) as an adjunct treatment for patients with antibiotic-associated diarrhea and was administrated to recipients via retention enemas (52). Despite the empiric success of the treatment, the etiology of post-antibiotic colitis (commonly termed today “pseudomembranous colitis”) remained unknown for nearly 20 years following that report when it was found that toxins from *C. difficile*, an anaerobic commensal bacterium, were responsible for the pathologic process (53, 54). Following these revelations a plethora of evidence of varying quality demonstrated the clinical effect that FMT has on pseudomembranous colitis, culminating with a landmark randomized clinical trial demonstrating the significant superiority that FMT has on recurrent *C. difficile* infection over the standard antibiotic treatment (55). This seminal study featured an overall 90% success rate of FMT as treatment of recurrent *C. difficile* infection and was terminated prematurely given these dramatic interim analysis results.

**Figure 1:**
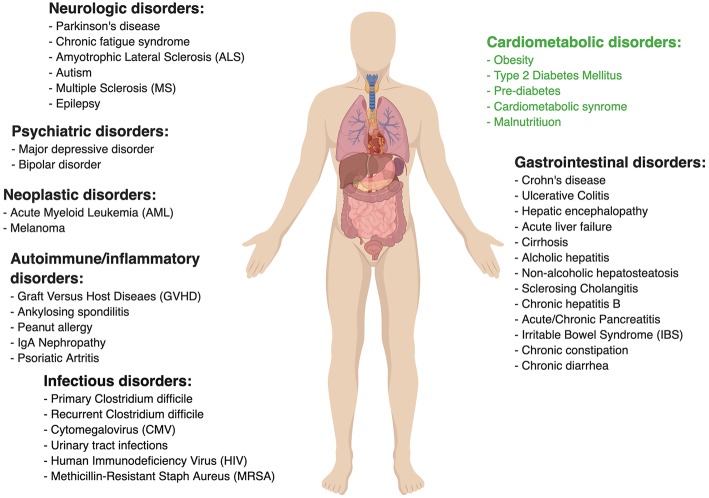
Ongoing clinical trials to evaluate fecal microbial transplant. Data taken from www.clinicaltrial.gov. Search words: fecal microbial transplant/FMT.

Figure 1 lists other medical conditions in which the efficacy of FMT is currently being clinically investigated. Many of the associated studies assessing these various indications are rather preliminary, thereby tending to be very heterogeneous in their design (i.e., inclusion criteria, treatment protocol, etc.). For example, FMT for Ulcerative Colitis has been tested in a few randomized controlled trials, some of which demonstrated clinical efficacy (56–58) while other studies failed to document such effect (59). FMT in Crohn's disease was evaluated mainly in small case series and has been proven to be more challenging, potentially because of pathophysiological differences from Ulcerative Colitis giving rise to technical difficulties (such as retention enema not reaching the site of active inflammation in small intestinal Crohn's disease). One study of 30 patients with refractory Crohn's disease noted promising results of 86.7% clinical remission in the first year following treatment and 76.7% remission rate in the second year (60), however, another study failed to reach such results (61).

Primary sclerosing cholangitis (PSC) is an auto-inflammatory disorder of the bile ducts and is associated with IBD, dysbiosis, and interrupted barrier function (62). A recent small uncontrolled clinical study in 10 PSC patients, has demonstrated FMT to improve bacterial diversity and Alkaline phosphatase (a disease-severity surrogate marker) levels, however, no other clinically meaningful disease parameters were reported to improve (63). IBS was also suggested to improve after FMT in a recent randomized controlled study including 90 patients, demonstrating that 65% of patients had symptomatic relief with FMT vs. 43% in the placebo group (*p* = 0.049) (64). Despite these encouraging results, a smaller scale randomized trial reported contradicting results favoring the placebo group (65), adding to the controversy surrounding FMT as a therapeutic measure in IBS. Considering these scarce evidence and in spite being microbiome-associated diseases, FMT in Crohn's disease, PSC, and IBS remains investigational as of now.

## FMT in Cardiometabolic Syndrome—Preclinical Research

Investigational use of FMT from mouse or human origin, transferred into germ-free (GF) mice which are completely devoid of a microbiome, has greatly advanced our understanding of the gut microbiome's causal roles in contributing and regulating cardiometabolic syndrome (Table 1). GF mice suffer of multiple metabolic alterations. Upon “conventionalizing” GF mice by transplantation of microbiota from regular wild-type mice they gain weight and their insulin sensitivity decreases back to normal levels (73). When GF mice are colonized with fecal microbiota from obese mice they gain even more weight and develop features of cardiometabolic syndrome, probably due to increased energy harvest from the diet (66, 72, 74). Some reports suggest that GF mice are resilient to diet-induced obesity by means of high-fat diet feeding (75–77), but others dispute these claims (78–83). These conflicting reports may stem from experimental differences in dietary macronutrients, namely fat/protein/fibers source and content (77, 84, 85).

**Table 1:** Gut microbiota modulation in cardiometabolic syndrome.

**Source**	**Subjects**	**Main findings**	**Strengths**	**Limitations**
**ADIPOSITY AND OBESITY**				
Turnbaugh et al. (66)	Humans Mice	Obesity is associated with a distinct microbiome with a high capacity to harvest energy from food.	Human microbiome functional analysis	Pre-clinical No mechanism proposed
Thaiss et al. (40)	Mice	A post-dieting associated microbiome that persists during successful dieting, contributes to post-dieting weight-regain in a flavonoid-dependent manner.	Metabolomic analysis Human microbiome functional analysis A mechanism is proposed	Pre-clinical
Ridaura et al. (67)	Humans Mice	Obesity is transferrable upon FMT from obese humans to mice. Co-housing recipients of obese microbiome with recipients of lean microbiome, prevented weight gain, and microbiome of all co-housed mice resembled “lean” configuration.	Weight discordant twins as controls	Pre-clinical No mechanism proposed
Tremaroli et al. (68)	Humans Mice	Obese humans who underwent bariatric surgery experienced long-term weight-loss and reduction in TMAO levels accompanied by microbiome composition shift. FMT to GF mice resulted in reduced adiposity.	Human samples Microbiome functional analysis Bile acid analysis	No different in net weight gain No insulin sensitivity assessment
**GLUCOSE METABOLISM**				
Reijnders et al. (69)	Humans	A 7 days course of either Vancomycin or Amoxicillin did not affect host metabolism in overweight or obese adults, despite altered microbial composition, after 8 weeks from treatment initiation.	Randomized placebo-controlled Double blinded−8 weeks follow-up	Short antibiotic exposure Small sample (*N* = 56)
Vrieze et al. (70)	Humans	A 7 days course of oral Vancomycin in metabolic syndrome patients decreased fecal microbial diversity and fecal secondary bile acids, increased plasma primary bile acids, and decreased insulin sensitivity. A 7 day course of Amoxicillin did not affect any of these parameters.	Randomized controlled trial	Short antibiotic exposure Single blinded Short follow-up (1 week) Modest effect size Significance was marginal Small sample (*N* = 20) No placebo No microbial functional analysis
Zeevi et al. (36)	Humans	Post-prandial glycemic response to different foods is individual and can be predicted based on clinical and microbial parameters.	Human study Large sample size (*N* = 900) Validation cohort	Use of stool samples Focused on glycemic response Microbiome contribution to glycemic prediction isn't clear
**HYPERTENSION**				
Li et al. (71)	Humans Mice	The gut microbiome of hypertensive patients is distinct, and hypertension is transferable upon FMT.	Large human cohort (*N* = 196) Metabolomic analysis	No mechanism proposed
**METABOLIC SYNDROME**				
Vijay-Kumar et al. (72)	Mice	TLR5 KO leads to hyperlipidemia, hypertension, insulin resistance accompanied by microbial composition shift and is transferrable to WT GF mice upon FMT.	Wide metabolic assessment	

*The table depicts a representative number of studies demonstrating that cardiometabolic syndrome features may be affected by gut microbial modulation. Only the main and most relevant findings, limitations, and strengths are presented*.

Another pre-clinical example of the potential utilization of FMT in metabolic disease involves essential hypertension, which is considered a common feature of cardiometabolic syndrome spectrum. Metagenomic and metabolomic analyses of stools from 99 individuals with hypertension in comparison to samples from 56 subjects with pre-hypertension and 41 healthy individuals revealed that the microbiome of pre-hypertension subjects was more similar to that of hypertensive patients, and was associated with decreased microbial diversity (71). When hypertensive patients' feces were transferred to GF mice, the blood pressure of recipient mice had increased in comparison to GF recipients of healthy donor microbiome. Elevation of blood pressure following FMT was also reported in conventional mice recipients (86). Altogether these results suggest an important role of the gut microbiota in hypertension development, however, the lack of human data and mechanistic explanations for such a role necessitates additional investigation.

**Figure 2:**
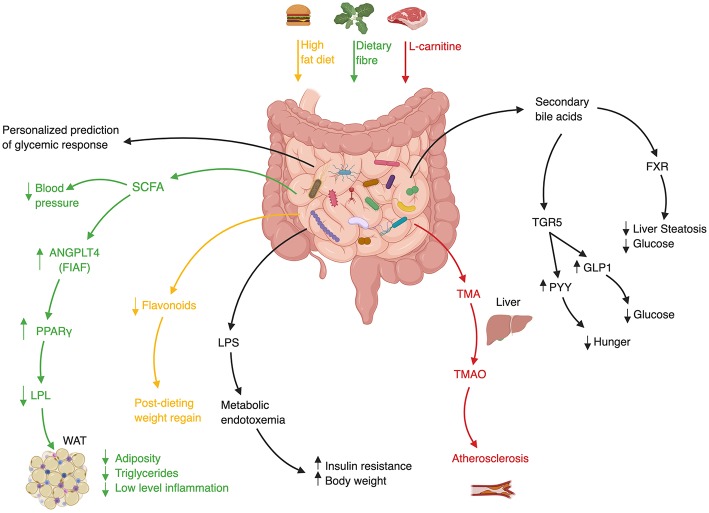
Mechanisms of Gut Microbiota involvement in Cardiometabolic Syndrome. ANGPLT4–Angiopoietin-like 4, FIAF–Fast Induced Adipose Factor, FXR–Farnesoid-X Receptor, GLP1–Glucagon-like Peptide 1, LPL–Lipoprotein Lipase, LPS–Lipopolysaccharide, PPARγ-Peroxisome proliferator-activated receptor γ, PYY–Peptide YY, SCFA–Short Chain Fatty Acid, TMA–Trimethylamine, TMAO–Trimethylamine-N Oxidase, WAT–White Adipose Tissue (36, 40, 91–93).

Likewise, GF mice fecal transfer experiments also suggest that the gut microbiome may modulate insulin sensitivity in various contexts (87, 88), weight gain (40), and fatty liver (40). Interestingly, this approach suggested a causal role of the gut microbiota in modulating the activity of Metformin, a medication used as first-line treatment for diabetes. In spite of this drug's extensive clinical use, the mechanism by which it increases insulin sensitivity remained elusive. Who et al. carried out a randomized placebo-controlled clinical trial of 4 months of Metformin vs. placebo in 40 treatment-naïve diabetic patients, to show that Metformin alters the gut microbiota (89). They further showed that the transfer of Metformin-altered human microbiota to GF mice improved recipients' insulin sensitivity. In a subsequent study Sun et al. utilized a metagenomic and metabolomic analysis to probe the mechanism behind microbiome-dependent metformin activity. They revealed that newly diagnosed diabetic patients who started treatment with metformin, experienced a decrease in *Bacteroides fragilis* abundance accompanied by an increase in a specific bile acid (glycoursodeoxycholic acid, GUDCA) (90). These changes were associated with inhibition of Farnesoid-X receptor (FXR) signaling, a receptor known to have a large impact on metabolic functions such as insulin sensitivity (Figure 2) (94). Both *B.fragilis* colonization and FXR knock-out abrogated Metformin's beneficial effect on insulin resistance. Finally, fecal microbial transplant from diabetic patients receiving Metformin improved insulin sensitivity in conventionally-raised antibiotic-treated mice compared to transplant from treatment-naïve diabetic patients. Altogether, these studies suggest that Metformin has a prebiotic quality (89, 90).

FMT of human microbiome configurations into GF mice was also used to show a causal role of the gut microbiome in mediating the beneficial effect of bariatric surgeries, which were originally designed to treat morbid obesity, but were also found to be extremely effective in also treating other cardiometabolic syndrome disorders such as insulin resistance and TIIDM (95–97). Results from studies utilizing rodent models of bariatric surgery have suggested a causal role of the gut microbiome in the metabolic beneficial effects of bariatric surgery (98–103). In one study, GF mice who were treated with fecal microbiome from previously obese patients who underwent bariatric surgery exhibited decreased adiposity compared with mice colonized with the microbiota of obese patients. In two additional studies, GF mice who received FMT from previously-obese rodents who underwent bariatric surgery also demonstrated weight loss and improved insulin sensitivity (100, 104), however, the observed improvements were very mild. Although these findings suggest a prebiotic mechanism of bariatric surgeries, the role of gut microbiota in post-bariatric surgery metabolic improvement warrants further inquiry. More importantly, revealing the mechanisms governing it is of special interest as it may lead to new therapeutic targets in cardiometabolic syndrome treatment as an alternative to a risky invasive procedure.

An optimized controlled methodology that served to disentangle genetic and environmental impacts on the microbiome while highlighting its impact on metabolic health involves characterization of the microbiome of siblings including twins, and their FMT into GF mice. Twins, sharing similar genetics and early-life environmental exposures, have relatively similar microbiome compositions (105, 106). One group performed FMT with microbiota from pairs of weight-discordant twins into separate groups of GF mice and measured their adiposity by quantitative magnetic resonance imaging (67). GF mice recipients of obese-twin microbiome developed increased adiposity independently of food intake. Interestingly, a repeat of the same experiment while co-housing recipients of microbiota from both lean and obese donors (in which coprophagy leads to equilibration of microbiome configuration) resulted in the loss of adiposity in both recipient groups, which under this equilibration setting were found to harbor a “lean” microbiome. These results of both transmissible adiposity phenotype of weight-discordant twins and lean-microbiome dominance upon co-housing were later confirmed by other independent groups (67, 107).

Preclinical data from FMT experiments into none-GF animals also support a significant role of the gut microbiome in cardiometabolic disease. Microbiome depletion by means of broad-spectrum antibiotics exposure improved insulin-resistance in conventional mice fed obesogenic diet (108). Rats fed with fructose-reach diet developed cardiometabolic syndrome that was improved upon FMT from rats fed normal chow (109). Resveratrol is a substance produced by plants in response to stress and is believed to have beneficial effects in cardiometabolic syndrome (110, 111). FMT from Resveratrol-treated donor mice improved insulin resistance in recipient mice (112). A recent study by the same group recapitulated the same phenotype with respect to insulin resistance and also showed improvement in hypertension in recipient mice (86). Interestingly, this effect was also evident when obese recipient mice were given heat-killed FMT, suggesting the presence of metabolites, non-bacterial microbes that survived heat killing, or non-viable bacterial components may mediate the beneficial effect. Strikingly, FMT from Resveratrol-treated mice was even more effective than direct Resveratrol supplementation.

With these encouraging preclinical data notwithstanding, data from preclinical models should always be treated with “a grain of salt” since very frequently preclinical evidence fails to be translated into advancements in human clinical care. Limitations in that regards include species specificity in the colonization of some microbial strains, differences in sample preparation and storage between studies, inherent physiological differences between mice and humans with regard to microbiome composition and function, bowel transit time, and other gastrointestinal physiological features.

## FMT in Cardiometabolic Syndrome—Clinical Research

Data to support the role of FMT in humans suffering from features of cardiometabolic syndrome is limited, but preliminary studies who mainly focused on one feature of cardiometabolic syndrome, insulin resistance, show promising preliminary results suggesting that FMT may favorably impact some metabolic features in humans. A landmark study randomly assigned 18 treatment-naïve male cardiometabolic syndrome patients to receive a single duodenal infusion of either allogeneic-FMT from a lean healthy donor or autologous-FMT, i.e., their own stool as placebo equivalent (14). Six weeks after the infusion peripheral insulin sensitivity improved only in recipients of healthy donor microbiota. This change was also accompanied by an increase in bacterial diversity, a measure of “microbiome quality,” and in butyrate-producing bacterial species who are suggested to exert a beneficial metabolic effect on mammalian hosts [Figure 2; (113, 114)]. The strengths of the study were the randomized double-blind design, the evaluation of microbial parameters before and after treatment, and the evaluation of these parameters in the small intestine which is often overlooked owing to technical difficulties. The main limitations of the study were the modest effect size of the investigated intervention, its small samples size and short-term follow-up, the inclusion of males only, the non-anaerobic preparation of donor's fecal samples which may have impaired their quality, and the use of a relatively shallow characterization of microbial composition without a functional metagenomic analysis. Nonetheless, this was the first randomized study in humans suggesting that the gut microbiome may improve insulin sensitivity.

In a sequel trial, the same group of investigators subsequently randomized 38 obese male patients with cardiometabolic syndrome to receive either allogeneic-FMT from lean donors (*n* = 26) or autologous-FMT [*n* = 12; (115)]. At 6 weeks following FMT, they observed a significant shift in both fecal and duodenal microbial composition, which was accompanied by an improvement in peripheral insulin sensitivity. Importantly, these changes were observed in only half of the treatment group while the other half did not exhibit either microbial shift or change in insulin sensitivity. Interestingly, at 18 weeks following FMT the recipients' fecal and duodenal microbial composition returned to baseline and so did their insulin sensitivity. This transient effect of FMT on insulin sensitivity supports a role of the gut microbiome in human insulin sensitivity. Further analysis revealed that response to lean-FMT was largely dependent on recipients' and not donors' baseline characteristics, and more specifically the recipients' initial microbiome diversity. Of note, the study featured a longer follow up of four and a half months, larger sample size and the comparison of single vs. double FMT administrations (no difference was found between them). This study highlights the complex issue of “colonization resistance” and raises the issue of whether it is driven by the endogenous microbiome (116) or the host's immune system (117), as well as the issue of donor selection which comprise a major hurdle in FMT-based therapy.

A third trial assessed the impact of FMT on atherosclerosis. Trimethylamine-N-oxide (TMAO) is a microbiome-related metabolite that was shown to be associated with atherosclerosis and increased cardiovascular risk (118–120). Red meat contains high amounts of L-carnitine which is converted by members of the gut microbiome to TMA, which after its absorption in the intestine is converted in the liver to TMAO (Figure 2). Interestingly, a vegan diet selects for bacteria with low capacity to metabolize L-carnitine, resulting in vegans having a lower amount of circulating TMAO (119). In a small randomized double-blind pilot trial carried out by the same group of investigators, TMAO production and vascular inflammation were evaluated, in 20 obese male patients suffering from cardiometabolic syndrome who received either allogeneic-FMT (*n* = 10) from a vegan donor or autologous-FMT [*n* = 10; (121)]. Although vegan-donor FMT induced a shift toward vegan-like gut microbiome in some but not all recipients, this shift was not translated into beneficial effects on surrogate markers of arterial inflammation and atherogenicity. Further clinical trials to evaluate the efficacy of FMT in improving insulin resistance and obesity are currently underway.

## Challenges and Limitations in FMT Implementation

This apparent lack of comprehensive reproducible evidence to the efficacy of FMT in most indications, including cardiometabolic disease, may stem from several conceptual and methodological reasons.

### Donor Selection and Preparation

Donor selection and preparation results from multiple studies suggest that responsiveness to FMT is, to a large extent, dependent on the donor, highlighting the importance of donor selection (Box 1). Selecting donors is a difficult task both because the gut microbiota is a complex entity making its quality-control challenging, and because infectious screening to prevent the transfer of communicable diseases from donor to recipient is costly, limited and debatable (122–125). Inter-individual differences in microbiome composition are vast and the interaction between the donor's microbial strains and metabolites and the recipient's endogenous microbiome and immune system remain elusive and may prevent effective colonization, stabilization, and function in an unpredictable manner. Monitoring the co-existence of donor and recipient strains following FMT recently revealed that new donor-derived strains are less likely to colonize the recipient's gut than strains that already exist in the recipient, shifting the focus from “donor-selection” to proper “donor-recipient matching” (117). In the very early days of modern FMT, recruited donors were mostly among family relatives of recipients, based on the hypothesis that shared environment and genetics promote similar microbial configuration which will facilitate colonization and thus improve treatment efficacy (126). However, although early reports favored family-relative donors (127, 128), more recent studies aimed directly to compare between related and unrelated healthy donor volunteers, showed no advantage to any group over the other (129–133). Since diet is known to be a major environmental influence on the gut microbiome, some investigators used a donor selecting strategy based on their diet (121).

Box 1 **“Super donors.”**

There is growing evidence that FMT therapeutic success depends on the microbial diversity and composition of the stool donor, leading to the concept of “super-donors”—a term suggested to describe donors whose stool is therapeutically significantly more effective when compared to other donors treatment outcomes (134). Currently, there is little but promising clinical evidence for the existence of super-donors, including one randomized clinical trial (56) investigating the efficacy of FMT for inducing clinical remission in patients with Ulcerative Colitis, where out of nine patients treated with FMT who achieved clinical remission, seven patients received stool from the same donor. This finding was seen in an additional randomized control trial by Paramsothy et al. when FMT that contained stool from one donor exhibited a higher remission rate compared with patients treated without the samples from the super-donor.

### Sample Handling

**Figure 3:**
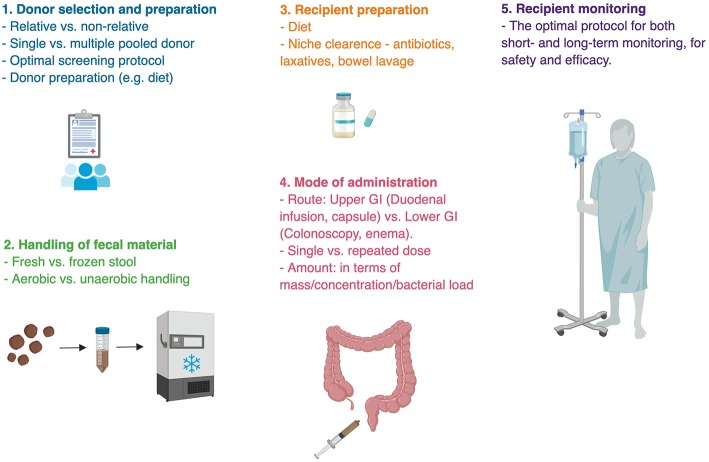
Challenges in fecal microbial transplant. Methodological uncertainties are present in almost every step of fecal microbial transplant. There is no single ideal protocol for FMT, rather different indications for FMT require appropriate methodology. Bullet points represent areas of uncertainty (135, 136).

Donated sample handling from collection to administration is an additional process that predisposes to methodological and outcome differences (Figure 3). After their collation samples are generally resuspended and diluted in isotonic fluids, filtered from solids, all the while being in an anaerobic condition. The samples are then either transplanted or stored frozen in a stool bank (Box 2) for later use to provide an on-demand availability. It remains unknown whether a dietary or antibiotic “pre-conditioning” of the donor enhances FMT's efficacy. Likewise, it remains unknown whether freezing compromises sample's quality (137). One remarkable small human study transplanted bacterial-free FMT, by filtering fecal samples prior to their transplantation, to successfully treat *C. difficile* infection, underscoring the importance of fecal metabolites and non-bacterial microbiome in FMT's efficacy, which necessitates further exploration (138, 139).

Box 2 **Stool banking.**

Since related FMT donors failed to show any benefit over unrelated donor FMT, healthy donor FMT became more available as unrelated donor FMT was more easily accessible and led to the establishment of stool banks (140). These banks aid treating physicians to initiate treatment instantly when needed, as FMT donors are recruited, evaluated, and screened for infectious disease in advance (141). So far, such banks exit only in several countries worldwide (142).

### Mode of Administration

Additional pending obstacles to broader utilization of FMT are the appropriate doses and route of administration [e.g., oral capsules, gastroduodenoscopy, colonoscopy, or enema; (143–146)]. The gut microbiome composition changes spatially throughout the length of the gastrointestinal tract (116, 147). It is generally assumed that the disease-associated portion of the gastrointestinal tract is the source of dysbiosis, and therefore should come in contact with donor's fecal material. For instance, trials for FMT in Ulcerative Colitis most often utilize colonoscopies and enemas (148), while trials looking at FMT for cardiometabolic syndrome, known to be affected by incretin secretion from the proximal gastrointestinal tract utilize duodenal infusions (14, 115, 121). However, it is possible and currently unexplored that combining more than one administration route is superior to a single administration route. In addition, repeated administrations might be needed to enable primary colonization or maintaining it in medical conditions that are chronic in nature (56, 59, 149). Indeed, an initial administration by colonoscopy followed by two subsequent enemas were successful at inducing remission in Ulcerative Colitis patients (57). These important methodological considerations may greatly impact clinical results of FMT and merit further consideration.

### Colonization Resistance

Many of the pre-clinical FMT models fail to translate into clinical practice (131, 150–154) while those that do mainly involve small-scale short-term clinical trials (57, 155–158). One potential caveat leading to FMT failure, which is often under-studied, includes colonization resistance of the recipient's gut to the transplanted ecosystem, which may differ between transplanted individuals. Such resistance has been recently demonstrated toward exogenous probiotics (116) and may involve intrinsic features of the indigenous microbiome, or of the host innate and adaptive immune responses. However, these donor-recipient-specific features may potentially enable to predict colonization success ahead of FMT, thereby suggesting that future personalized FMT optimization and personalized tailoring may be feasible (116).

### Adverse Effects

Although reported to be fairly safe in most clinical trials, FMT is not free of adverse effects, and mild temporary adverse effects are quite common. These include mild diarrhea (reported in up to 94% of cases in some series), abdominal pain (31%), abdominal bloating, nausea, headaches, and fatigue (159). Although most patients' complaints resolve within a few hours from treatment administration, some patients suffer from prolonged symptomatology. Some reports linked FMT with IBD worsening (160). A case report described new-onset obesity following FMT from an overweight donor in a previously lean patient treated for *C. difficile* (161). Furthermore, one study with a fairly long follow up examined whether patients developed new conditions following FMT and found that 5.1% of patients developed immunologic diseases, including peripheral neuropathy, Sjogren's disease, idiopathic thrombocytopenic purpura, and rheumatoid arthritis (162). Whether these are directly caused by transferred fecal elements or are coincidental findings remain unknown. One patient has died due to aspiration during sedation for FMT via colonoscopy, and another patient died during duodenal infusion of FMT (163).

Additionally, as stool contains thousands of bacterial strains, viruses, fungi, parasites, and a vast array of metabolites, FMT poses a constant risk of microbial transfer to donors that may harmfully impact them. Pathogenic screening of donor samples is often very limited and includes common transmissible pathogens (e.g., *Cytomegalovirus, Epstein-Barr virus, Hepatitis A, B, C, and E, Norovirus, Rotavirus, Syphilis, Entamoeba histolytica, Strongyloides stercoralis, Giardia lamblia, Cryptosporidium parvum, Blastocystis hominis, Helicobacter pylori, Salmonella, Shigella, Yersinia, E. coli O157:H7, Vancomycin-resistant Enterococcus, methicillin-resistant Staphylococcus aureus, C. difficile, Campylobacter, Vibrio cholera, Listeria monocytogenes*), while overlooking unknown transmissible pathogens or commensals that may impact recipient physiology in the short or long-term period (163). Indeed, several cases have emerged in which FMT was suggested to contribute to unrelated diseases in recipients (161, 164, 165). Albeit unproven, these potentials highlight the non-specific and undefined nature of the transferred microbial configuration. To this end, the use of rationally-defined bacterial consortia (166–168) and autologous microbiota transplantation (Box 3) are holding great promise as possible future refinements of FMT toward safer therapeutic options. Consideration and understanding of the scope of adverse effects of FMT are greatly needed, in assessing risk vs. benefit and ensuring a safe and efficacious procedure.

Box 3 **Autologous FMT.**

Although often used as placebo-equivalent in allogeneic-FMT clinical trials, autologous FMT might have a beneficial value in certain clinical situations. Autologous-FMT involves using stool taken from patients at an earlier time point while in a disease-free state or before a critical procedure (169, 170). In this context, the recipient serves as his/her own donor. Treating a patient with his own stool taken during a healthier, relatively homeostatic-state, aims at replenishing his original “healthy” microbiota, and by that restoring some aspects of his normal physiologic state. This strategy lacks many of the disadvantages that allogeneic-FMT has such as donor selection, colonization resistance, and unrelated pathogenesis. Interestingly, a single infusion of autologous-FMT was shown to not only rapidly and effectively reconstruct patients' original microbiota composition to its baseline, but also to restore the human epithelial transcriptome throughout the entirety of their gastrointestinal tract, following antibiotic perturbations (171, 172). Although microbiome parameters and host's global gut gene expression profile were shown to be swiftly reconstituted by autologous-FMT following antibiotics exposure, clinical benefits of this approach merit further studies (171, 173). Examples of currently ongoing clinical trials utilizing autologous-FMT include trials testing its efficacy in treating acute graft-vs.-host disease and as prevention of *C. difficile* infection following hematopoietic stem-cell transplantation which is often associated with high risk for *C. difficile* infection due to exposure to multiple courses of antibiotics.

### Cost Effectiveness

In addition to the therapeutic value of FMT, several cost-effectiveness analyses assessed the cost-effectiveness of FMT in recurrent *C. difficile* infection. The first demonstrated a clear advantage to FMT delivered by colonoscopy as compared to other treatment strategies in patients with recurrent *C. difficile* infection (174), but only included a single FMT infusion. The second demonstrated a cost-effectiveness advantage of FMT in the third *C. difficile* colitis recurrence (175). The third compared oral vancomycin to two methods of FMT deliverance (nasoduodenal and colonoscopy) of FMT, showing that both methods are superior to oral antibiotics (176). Other studies that analyzed the cost-effectiveness of FMT for the treatment of initial *C. difficile* infection, failed to demonstrate that FMT has an advantage over oral metronidazole (177, 178).

### Cardiometabolic Syndrome-Specific Limitations in FMT Utilization

On top of the above inherent limitations and challenges of FMT in general (Figure 3), there are a few barriers to the utilization of FMT in treating cardiometabolic syndrome. First, despite robust preclinical evidence, human evidence to support FMT in cardiometabolic syndrome is fairly scarce and weak. All three clinical trials were carried out by the same group of researchers and demonstrated modest, transient effect in a limited selection of patients, with considerable variability in response to treatment. Second, different professional organizations define cardiometabolic syndrome using different sets of criteria, with no single universal set of diagnostic criteria accepted by all (1, 179, 180). Moreover, all definitions require patients to only partially fulfill the list of criteria, therefore patients have different metabolic aberrations of variable severity, resulting in a very heterogeneous patients' population. Last, cardiometabolic syndrome is a chronic disorder that may necessitate multiple fecal transfers, in avoiding a transient effect. This repeated regimen is expected to lower its cost-effectiveness and patients' compliance.

## Prospects and Conclusions

While FMT has emerged as an important therapy for defined indications such as *C. difficile* colitis, it faces major barriers and challenges in being adopted as an intervention in cardiometabolic disease. With that said, a variety of challenges may be tackled in optimizing FMT for chronic metabolic indications. Issues such as donor selection, sample handling, predicting recipient compatibility to a given donor microbiome, and standardization of therapeutic FMT protocol may result in improved outcomes of these interventions and in higher reproducibility between studies. Moreover, a better understanding of causative vs. “passenger” bacteria, and of the contribution of non-bacterial components of the gut microbiome such as the virome and fungome may enable to formulate a defined microbial signature that would optimize efficacy while improving the safety of the procedure and its long-term effects on the recipients.

Utilizing autologous-FMT as means of “rejuvenating” a person's microbiome toward a pre-disease configuration may offer an attractive opportunity to optimize colonization, ensure safety, and avoid inter-individual incompatibilities. It may prove clinically useful in treating some complications of dysbiosis-inducing elective medical interventions such as chemotherapy (181), hematopoietic stem-cell transplantation (171, 182), surgery (183) or exposure to wide-spectrum antibiotics (172, 184, 185). In the context of cardiometabolic syndrome it may help to reverse microbiome contributions toward obesity, TIIDM, and NAFLD while resetting the host toward a healthier metabolic state (40).

While a deeper mechanistic understanding of discrete commensal functions and their modulatory activities on bioactive metabolites may enable the development of more precise microbiome-associated treatments, the advantage of patient supplementation with an intact bacterial ecosystem with its intricate and cross-supportive interaction networks may enhance the clinical efficacy of such treatment over mono-inoculation with a commensal or its metabolic product. However, adverse effects associated with the unknown components of the transferred ecosystem into a foreign host need to be extensively studied and taken into consideration when assessing the risk vs. benefit of FMT for metabolic and other indications.

## Author Contributions

AL and NH contributed equally to literature search, integration of data, and the writing of this review. EE supervised data gathering and integration and headed the writing of the review.

### Conflict of Interest Statement

EE is a paid consultant at DayTwo and BiomX. The remaining authors declare that the research was conducted in the absence of any commercial or financial relationships that could be construed as a potential conflict of interest.
